# Editorial: A Revised Approach to the Pathogenesis and Diagnosis of Myelodysplastic Syndromes With Therapeutic Implications

**DOI:** 10.3389/fonc.2018.00261

**Published:** 2018-07-17

**Authors:** Carmen Mariana Aanei, Lydia Campos Catafal, Denis Guyotat

**Affiliations:** ^1^Laboratoire d’Hématologie, CHU de Saint-Etienne, Saint-Etienne, France; ^2^Département d’Hématologie et Thérapie Cellulaire, Institut de Cancérologie Lucien Neuwirth, Saint-Etienne, France

**Keywords:** myelodysplastic syndromes, innate and specific immunity, inflammatory microenvironment, stromal microenvironment, flow cytometry for myelodysplastic syndromes diagnosis

**The Editorial on the Research Topic**

**A Revised Approach to the Pathogenesis and Diagnosis of Myelodysplastic Syndromes With Therapeutic Implications**

In 1938, Cornelius Packard Rhoads and his colleague William Halsey Barker from the Hospital of the Rockefeller Institute for Medical Research in New York described the first cases of what they termed “refractory anemia” in patients with acquired anemia and refractory to treatment with iron and liver extract. Since then the most common method of investigation has been the cytological evaluation of the bone marrow (BM) aspirates [reviewed in Ref. (1)]. Over time, it has been shown that this method is highly subjective and dependent on the pathologist’s experience, and that it cannot detect the disease in the early stages.

For these reasons, in 2001 when in *Blood* (2) Maryalice Stetler-Stevenson published the first panel for multicolor flow-cytometry (MFC) evaluation of BM aspirates in cytopenic patients with suspected myelodysplastic syndromes (MDS), it was first welcomed as a major step forward. However, the benefits of applying this method have proven to be limited, despite the development and the improvement of various scoring systems [e.g., Kern score (3), Ogata score (4), Red score (5), etc.]. The recently published WHO classification stipulates that MFC findings alone are not sufficient to establish a primary diagnosis of MDS in the absence of conclusive morphological and/or cytogenetic data (6).

However, recent years have seen advances in MDS diagnosis. Emerging technologies in molecular biology, such as next-generation sequencing, have enabled identification of mutations associated with MDS (7) and of the molecular basis for resistance to azacitidine therapy (8). In addition, studies exploring the BM microenvironment have shown that inflammation is the underlying mechanism for the medullary abortion of hematopoietic stem progenitor cells (HSPC) and that the immune-mediated mechanism is responsible for the clonal evolution of the disease (reviewed in Sallman et al.).

Nevertheless, many questions remain open in this field. For example, how can the detection of an MDS-associated mutation help establish diagnosis in cases with no obvious clinical presentation and morphology? How can MFC help in the detection of clonal hematopoiesis or maturation abnormalities? How does the BM microenvironment contribute to the initiation and progression of the disease?

This research topic aims to bring to the attention of readers the recently proposed hypotheses for the underlying mechanisms in MDS and to highlight the utility of MFC for MDS diagnosis. It consists of five articles, including two original research articles and three reviews, elaborated by 18 authors holding different views on MDS pathogenesis and diagnosis.

Sallman et al. provide a comprehensive overview of the role of inflammation in MDS pathogenesis. The alarmin S100A8/9-primed microenvironment mediates the HSPC death by pyroptosis and is responsible for the propagation of the MDS clone through the assembly and activation of the redox-sensitive NLRP3 inflammasome and β-catenin, as well as by promoting mutations in HSPC founder genes.

Lambert et al. highlight the role of innate and specific immunity in BM immune surveillance and protection against MDS clone evolution and progression toward secondary leukemia. The pathological HSPC clones can take over the immune system by producing immuno-modulatory cytokines (IL-4, IL-10, and TGFβ) and growth factors (VEGF, TNFα, thrombopoietin, etc.) and by modulating Treg cell activity. In addition, the immune system itself can suffer from MDS progression due to central cytopenia installment, Treg accumulation, exhaustion of phagocytosis and cytotoxicity, repertoire skewing, and possible autoimmune disorders (Lambert et al.).

Wu et al. provide evidence that the stromal microenvironment is fundamentally altered in low-risk MDS (LR-MDS). The mesenchymal stromal cells (MSCs) in LR-MDS are characterized by a remarkable deficit in focal adhesion kinase that is downregulated and hypo-activated in this setting. In addition, MSCs in LR-MDS show impaired growth and clonogenic capacity and have a higher propensity to pro-adipogenic differentiation and attenuated osteogenic capacity. Along with reduced expression of SDF-1, these MSC abnormalities might be responsible for creating an unfavorable microenvironment for hematopoiesis.

Aanei et al. summarize the advantages and limitations of different approaches currently used in flow cytometry for MDS diagnosis. The authors stress the need to include criteria for BM hemodilution evaluation in order to avoid misinterpretation of antigen expression patterns or of maturation profiles. In addition, the rating of the contribution of each phenotypic marker used in MDS panels and identification of those which are non-qualifying might improve the various screening scores currently used for MDS diagnosis.

Picot et al. show that the monocyte subset evaluation by MFC, due to its simplicity and robustness, provides relevant information for CMML diagnosis. The authors suggest a ranking of biological tests for CMML diagnosis, first relying on the MFC test of blood samples that may exclude non-CMML patients from further invasive procedures. The NGS test and complete evaluation, including BM exploration, should be reserved for patients with abnormal MFC results.

## Author Contributions

CA, LC, and DG are co-editors of this research topic.

## Conflict of Interest Statement

The authors declare that the research was conducted in the absence of any commercial or financial relationships that could be construed as a potential conflict of interest.
